# Mirk/Dyrk1B Kinase Inhibitors in Targeted Cancer Therapy

**DOI:** 10.3390/pharmaceutics16040528

**Published:** 2024-04-11

**Authors:** Nikolaos Kokkorakis, Marios Zouridakis, Maria Gaitanou

**Affiliations:** 1 Laboratory of Cellular and Molecular Neurobiology-Stem Cells, Hellenic Pasteur Institute, 11521 Athens, Greece; nkokkorakis@pasteur.gr; 2 Division of Animal and Human Physiology, Department of Biology, National and Kapodistrian University of Athens, 15784 Athens, Greece; 3 Structural Neurobiology Research Group, Laboratory of Molecular Neurobiology and Immunology, Hellenic Pasteur Institute, 11521 Athens, Greece; mzouridakis@pasteur.gr

**Keywords:** Mirk/Dyrk1B kinase, cancer, cancer stem cells, quiescence, apoptosis, targeted cancer therapy, inhibitors, X-ray crystal structures

## Abstract

During the last years, there has been an increased effort in the discovery of selective and potent kinase inhibitors for targeted cancer therapy. Kinase inhibitors exhibit less toxicity compared to conventional chemotherapy, and several have entered the market. Mirk/Dyrk1B kinase is a promising pharmacological target in cancer since it is overexpressed in many tumors, and its overexpression is correlated with patients’ poor prognosis. Mirk/Dyrk1B acts as a negative cell cycle regulator, maintaining the survival of quiescent cancer cells and conferring their resistance to chemotherapies. Many studies have demonstrated the valuable therapeutic effect of Mirk/Dyrk1B inhibitors in cancer cell lines, mouse xenografts, and patient-derived 3D-organoids, providing a perspective for entering clinical trials. Since the majority of Mirk/Dyrk1B inhibitors target the highly conserved ATP-binding site, they exhibit off-target effects with other kinases, especially with the highly similar Dyrk1A. In this review, apart from summarizing the data establishing Dyrk1B as a therapeutic target in cancer, we highlight the most potent Mirk/Dyrk1B inhibitors recently reported. We also discuss the limitations and perspectives for the structure-based design of Mirk/Dyrk1B potent and highly selective inhibitors based on the accumulated structural data of Dyrk1A and the recent crystal structure of Dyrk1B with AZ191 inhibitor.

## 1. Introduction

Cancer therapies include surgery, chemotherapy, radiation, immunotherapy, hormone therapy, and targeted drug therapy. Targeted cancer therapy is directed against cancer-specific molecules and signaling pathways and thus has less non-specific toxicity compared to chemotherapy. Protein kinases are an important class of therapeutic targets for several human diseases, especially for human cancers because their dysregulation is implicated in tumorigenesis. Thus, exploring the role of oncogenic protein kinases has helped advances in cancer biology and led to targeted drug therapy and personalized medicine [1,2,3,4]. During the last years, there has been an increased effort in the discovery of selective and potent inhibitors for targeted cancer therapy, which benefit patients since they provide an improvement in tolerability compared to cytotoxic chemotherapies. The potential benefit of kinase inhibitors in cancer therapy depends on their high selectivity and potency against their targets, as well as on their pharmacokinetic (PK) and pharmacodynamic (PD) properties [5,6,7,8]. The discovery of oncogenic kinases and the complete mapping of the human kinome has increasingly pushed the research into the development of new anti-cancer drugs. Imatinib (STI571, Gleevec) was the first therapeutic protein kinase inhibitor that targets Bcr-Abl kinase in chronic myeloid leukemia (CML) [9]. Until 2018, there were 166 FDA-approved oncologic drugs, including small molecules, antibodies (mAbs), and proteins. Notably, small molecules interacting with protein kinases represent 28% of this total in the major category [5]. In 2023, there were 72 FDA-approved inhibitors targeting different protein kinases for the treatment of neoplasms (57 against solid tumors such as breast, lung, and colon, 11 against non-solid tumors such as leukemia, and 4 against both solid and non-solid tumors) [7,8].

Several protein kinase inhibitors that have been approved by the FDA to date target Bcr-Abl kinases in chronic myeloid leukemia; epidermal growth factor receptors (EGFRs) in non-small cell lung and breast cancer; vascular endothelial growth factor receptors (VEGFRs) in renal cell carcinoma and hepatocellular carcinoma; B-Raf (BRAF) kinase in advanced metastatic melanoma; MEK kinases in BRAF V600E mutant metastatic melanoma; anaplastic lymphoma kinase (ALK) in lung adenocarcinoma; Bruton tyrosine kinase (BTK) in B cell malignancies and lymphoma; and JAK2 kinase, phosphoinositide 3-kinase (PI3K), and cyclin-dependent kinases 4/6 (CDK4/6) in metastatic breast cancer [6,7,8].

Dual-specificity tyrosine phosphorylation-regulated kinase 1B (Dyrk1B), also known as minibrain-related kinase (Mirk) is a member of the DYRK kinase family. DYRKs comprise a family of protein kinases involved in several signal transduction pathways critical for development and cell homeostasis. DYRKs belong to the CMGC group of proline-directed serine/threonine kinases that are characterized by their ability to phosphorylate tyrosine, serine, and threonine amino acid residues [10,11,12]. DYRKs acquire their catalytic activity, upon their auto-phosphorylation at the second tyrosine residue of a conserved YxY motif, located at their activation loop, losing their ability to phosphorylate tyrosine residues on their substrates, while retaining their Ser/Thr phosphorylation activity [11]. Especially, a characteristic sequence motif DYRK homology box (known as the DH box), which is located at the N-terminal of the catalytic domain, supports the auto-phosphorylation of the conserved tyrosine during the maturation of the catalytic domain [10,11]. DYRK family members have been found in all eukaryotes and constitute an evolutionarily conserved family of protein kinases, which are key players in the regulation of cell cycle and differentiation, transcription, protein stability, and apoptosis, through the phosphorylation of several target proteins [10,12,13]. The mammalian DYRK kinase family comprises five members: the Dyrk I class, which includes Dyrk1A and Dyrk1B, and the Dyrk II class, which includes Dyrk2, Dyrk3, and Dyrk4 [10,11,12]. Recently, Dyrk1A and Dyrk1B have been evaluated as therapeutic targets for neurodegenerative diseases [14] and cancer [15,16], respectively. The human *Dyrk1A* gene is located on chromosome 21 in the Down syndrome critical region and is associated with Down syndrome and neurodegenerative diseases [14,17], whereas the human *Dyrk1B* gene is located on chromosome 19q13.2, a region often amplified in ovarian and pancreatic cancer [18].

Dyrk1B plays a critical role in myogenesis [19,20,21], spermatogenesis [22], adipogenesis [23,24,25,26], and neurogenesis [27,28,29,30], and it is implicated in human diseases, such as metabolic syndrome [23,24,25,26] and cancer [16,18,31,32,33,34,35,36,37]. Dyrk1B exerts its functions through its regulatory effects on cell cycle progression, cell differentiation, cell survival, cell motility, transcription [12,13], and inflammation [38,39]. Moreover, Dyrk1B comprises a central mediator of Sonic hedgehog/Gli [29,40,41,42,43], PI3K/mTOR/AKT [42,44], and RAF/MEK/ERK [45,46] signaling pathways in cancer. Dyrk1B is normally expressed at high levels in skeletal muscle and testis, with increased relative expression in cardiac muscle and brain compared to other normal tissues [19,47]. Dyrk1B was found to be overexpressed in various solid tumors and cancer cell lines, acting as a G0/G1 checkpoint kinase [16,48], maintaining the quiescence of cancer cells [16,18] and their viability under normoxic or hypoxic conditions [18,49], and as a tumor survival factor [16,35,37,45,50,51], thus conferring chemoresistance in cancer cells [52]. In particular, Dyrk1B is highly expressed in colorectal [15,31,53], prostate [54,55,56], and lung cancer [45,50]; pancreatic ductal adenocarcinomas [32,57,58,59,60]; rhabdomyosarcomas [61]; osteosarcomas [62]; and liposarcomas [63]. In addition, overexpression of Dyrk1B has been reported in breast [37,64,65], ovarian [18,33,35,36], and cervical cancer [66] and in melanoma [67].

Recently, Dyrk1B has emerged as a novel therapeutic target for cancer. Here, we review the research that has demonstrated Dyrk1B to be a valuable therapeutic target in cancer, and we refer to efforts and recent advances in the field of medicinal chemistry aimed at generating potent and highly specific Dyrk1B inhibitors exhibiting eliminated off-target effects against other kinases and especially its closely related member Dyrk1A. Performed studies were mainly based on homology models due to the (until recently) unknown Dyrk1B crystal structure. Moreover, we refer to DYRK family structure and crystallography studies with emphasis on those of Dyrk1A. Remarkably, the Dyrk1B crystal structure in complex with its potent and specific inhibitor AZ191 was submitted to the Protein Data Bank (PDB Entry: 8C2Z) while this manuscript was under preparation. 

## 2. Mirk/Dyrk1B Kinase Is an Emerging Therapeutic Target in Cancer 

Conventional chemotherapy targets rapidly dividing cells by blocking essential phases of cell division, such as mitosis and DNA synthesis, or by taking advantage of the susceptibility of proliferating cells to genotoxic and metabolic stress. A significant portion of cancer cells exist in quiescence, a reversible non-dividing state that makes quiescent cancer cells resistant to chemotherapy. Thus, a strategy for quiescent cancer cells that are resistant to chemotherapy is to re-enter the cell cycle. The regulation of the cell cycle by Dyrk1B is mediated by the phosphorylation of cyclin D1 [27,28,48,68] and cyclin-dependent kinase inhibitor p27^Kip1^ [69] promoting their degradation and stability, respectively. Additional regulatory mechanisms of the cell cycle have been reported for Dyrk1A/B kinases through the DREAM complex. Dyrk1A/B kinases activate the DREAM complex by phosphorylating the MuvB subunit LIN52 at the Ser28 residue [16,70]. Given that cancer cells require active Dyrk1B kinase to remain in a G0 quiescent state, the pharmacological inhibition of Dyrk1B is a possible therapeutic strategy to overcome the chemo- and radio- resistance of quiescent cancer cells [59,66].

Moreover, Dyrk1B acts as a prosurvival factor in cancer. RNAi-mediated depletion of Dyrk1B or its pharmacological inhibition diminishes cell survival and induces apoptosis in many cancer cell lines [34,36,50,57,60,62], as reflected by the increased intracellular levels of reactive oxygen species (ROS) [58,71] that are accompanied with DNA damage, indicated by phosphorylation of histone 2AX (H2AX) [72]. Moreover, Dyrk1B up-regulates the expression of several antioxidant genes, e.g., superoxide dismutases 2 and 3 (*SOD2*, *SOD3*) and ferroxidase in cancer cell lines [58,71]. The increased expression of antioxidant genes may be a mechanism of cancer cells to maintain higher ROS levels than normal cells and thus have increased sensitivity to further ROS accumulation. Hence, it has been proposed as a potential strategy for anticancer therapies targeting antioxidant mechanisms of cancer cells and the subsequent increase in intracellular cellular ROS levels [73].

**Figure 1:**
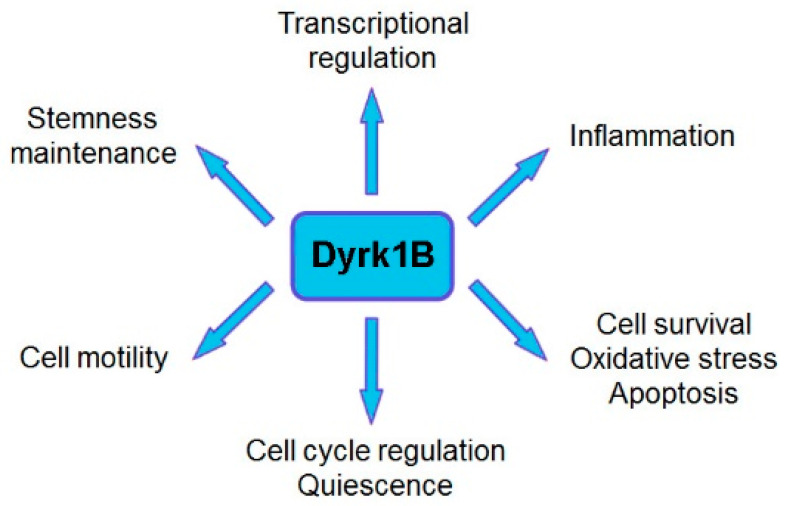
Summary of the major known functions of Dyrk1B. Dual-specificity tyrosine-regulated kinase 1B (Dyrk1B) plays a critical role in many biological processes in development and human disease, by regulating cell cycle progression/exit and quiescence, stemness maintenance, transcriptional regulation, cell motility, as well as cell survival, oxidative stress, and apoptosis.

**Figure 2:**
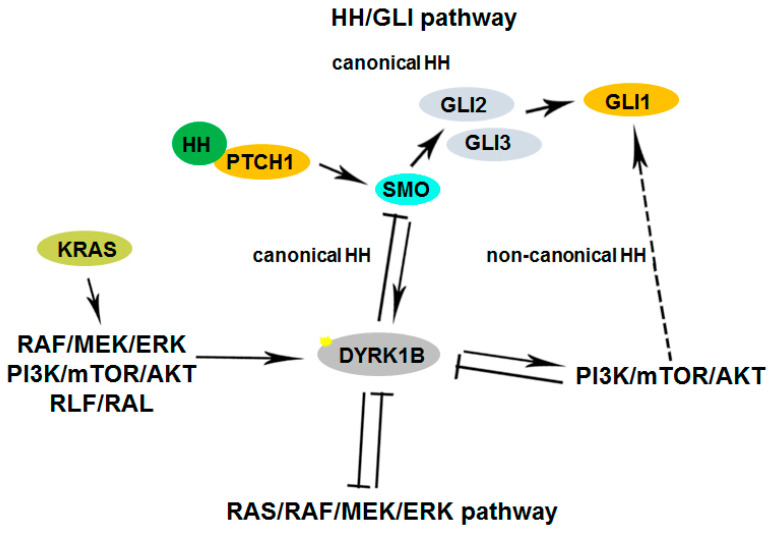
Cross-talk of Dyrk1B in signaling pathways in development and disease. Serum mitogens down-regulate Dyrk1B through the RAS/RAF/MEK/ERK signaling pathway. In turn, Dyrk1B is implicated in glucose homeostasis through the inhibition of the RAS–RAF–MEK pathway in metabolic syndrome which is accompanied by diabetes. In cancer, Dyrk1B is involved in a complex cross-talk with Hedgehog (Hh). Dyrk1B inhibits ‘canonical’ Hh signaling initiated by Smoothened, while it promotes ‘non-canonical’ Hh signaling and oncogenic Gli1 activity by promoting PI3K/mTOR/AKT signaling and PI3K–AKT-mediated stability of the Gli1 transcription factor. Conversely, activated AKT directly inhibits the expression of Dyrk1B. In cancer, oncogenic mutant RAS (KRAS) initiates the ‘non-canonical’ HH pathway through the activation of Dyrk1B, via an elusive mechanism, employing several RAS effectors such as RAF/MEK/ERK, PI3K/AKT, and RLF/RAL. Dashed lines represent indirect mechanisms and yellow star represents phosphorylation HH: Hedgehog; ERK: Extracellular signal-regulated kinases; SMO: Smoothened.

Furthermore, Dyrk1B maintains the quiescence of cancer stem cells (CSCs) [74] and regulates their maintenance under normoxic or hypoxic conditions. In normoxia, oxygen-sensing prolyl-hydroxylase (PHD1) activates Dyrk1B, which inactivates ID2 protein by phosphorylating it at residue threonine 27 [49], making it unable to displace the VHL-associated protein Cullin-2 (Cul2) component from the VCB–Cul2 ubiquitin ligase complex. The active VCB–Cul2 ubiquitin ligase complex promotes the degradation of hypoxia-inducible factor HIF2α that is required for the maintenance of cancer stem cells (CSCs) [49]. Conversely, hypoxic conditions lead to inactivated PHD1 and Dyrk1B, resulting in HIF2α stabilization [49]. The HIF2α stabilization facilitates CSC maintenance increasing the aggressiveness of human hypoxic brain tumors [49]. In glioblastoma cell lines, under hypoxic conditions, ID2 positively modulates HIF2α activity. Conversely, under normoxic conditions, the elevated expression of Dyrk1B leads to HIF2α destabilization, loss of glioma stemness, inhibition of tumor development, and a more favorable outcome for patients [49]. In addition, Dyrk1B cross-talks with the Hedgehog/Gli pathway, by inhibiting ‘canonical’ and enhancing ‘non-canonical’ Hh signaling, are implicated in cancer [40,41,42]. Dyrk1B functions and cross-talk with Hedgehog/Gli, PI3K/mTOR/AKT, and RAF/MEK/ERK signaling pathways implicated in cancer are summarized in Figure 1 and Figure 2, respectively.

## 3. Mirk/Dyrk1B Kinase Inhibitors 

### 3.1. AΖ191 Inhibitor

**Figure 3:**
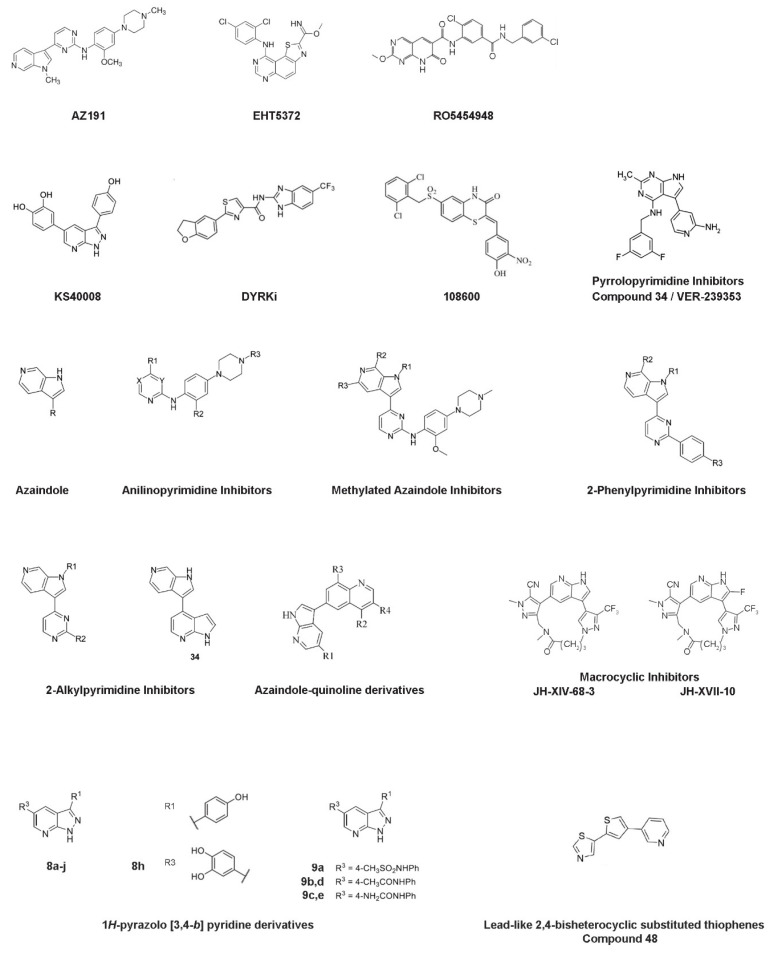
Chemical structures of the Mirk/Dyrk1B kinase inhibitors. Chemical structures of known Dyrk1B inhibitors that are referred to this review. Notably, the VER-239353 inhibitor (compound **34**) that belongs to pyrrolopyrimidine inhibitors [79] is different from the compound **34** that belongs to 2-alkylpyrimidine inhibitors [80].

AZ191 was identified in a protein kinase drug discovery project of AstraZeneca among a set of 6-azaindoles and was found to exhibit potent and selective inhibition of Dyrk1B in a kinase-selectivity screening (Figure 3). In particular, AZ191 inhibited Dyrk1B kinase activity in a cell-free assay with an IC_50_ of 17 nM, displaying about 5- and 110-fold increased selectivity over Dyrk1A (IC_50_ of 88 nM) and Dyrk2 (IC_50_ of 1890 nM), respectively [68]. Following its discovery, several studies have used AZ191 in Dyrk1B targeted cancer therapy combined or not with other kinase inhibitors and/or chemo- and radio-therapy approaches [46,63,67,68,75,76,77,78].

Oral squamous cell carcinoma (OSCC) is an aggressive invasive head–neck malignancy with a 5-year survival rate of <50%. It is associated with recurrence and metastasis. In a recent study [81] aiming to eliminate quiescent cancer stem cells (CSCs), a combinatorial drug approach was applied in primary OSCC cultures derived from patients. In particular, Dyrk1B, topoisomerase II, and histone deacetylase were targeted with AZ191, mitoxantrone (MX), and mocetinostat (MO) inhibitors, respectively. The effect of double treatments by using AZ191 and MX or AZ191 and MO inhibitors, respectively, promoted cancer cells to exit the quiescence state. Moreover, combined co-treatments with AZ191/MX or AZ191/MO resulted in CSCs with decreased glutathione levels, increased ROS levels, increased DNA damage, and increased mitochondrial stress, as indicated by elevated levels of cytochrome c [81]. These results provide a combinatorial therapeutic approach to target and minimize the quiescence and viability of oral CSCs simultaneously [81].

Dyrk1B is highly expressed in triple-negative breast cancer (TNBC) and is essential for TNBC cell proliferation and mobility. TNBC patients exhibit high expression of Dyrk1B and poor survival. Also, Ccdc97 and Znf581 were positively correlated with Dyrk1B expression and might be involved in Dyrk1B-mediated tumor malignancy in patients with TNBC, suggesting Dyrk1B as a potential therapeutic target for TNBC. Silencing or inhibition of Dyrk1B by AZ191 decreased the viability of TNBC cells by arresting the cell cycle at the G1 phase and reducing metastatic characteristics, such as migration and invasion. These results support Dyrk1B as a potential target in TNBC therapy [65].

Liposarcoma is a soft tissue sarcoma accounting for 20% of all sarcoma cases. Conventional chemotherapeutic agents have limited efficacy in patients with liposarcoma. Higher Dyrk1B expression levels in liposarcoma are correlated with a poor prognosis. Dyrk1B silencing by RNA interference or Dyrk1B inhibition by AZ191 inhibitor suppressed tumor growth, decreased cell motility and migration, and induced apoptosis in the liposarcoma cell lines SW872 and SW982 [63]. In addition, combinatorial treatment of SW872 and SW982 cells with AZ191 and doxorubicin resulted in increased cytotoxicity as detected by MTT assay, suggesting that Dyrk1B inhibition enhances the anti-cancer effects of doxorubicin [63].

Hepatocellular carcinoma (HCC) is the most prevalent cancer without effective treatment. The proliferative and oncogenic trophinin-associated protein (TROAP) has an essential role in several malignancies such as prostate cancer, gastric cancer, colorectal cancer, and HCC [82,83,84,85,86]. TROAP dramatically drives HCC cell growth, increasing the malignant proliferation of HCC cells in vitro and in vivo, leading to poor survival of patients with HCC. TROAP exerts its oncogenic function in HCC through its interaction with Dyrk1B. TROAP switches Dyrk1B activity by direct bounding, resulting in the cytoplasmic translocation of Dyrk1B, thus promoting cell cycle progression via activation of Akt/GSK-3β/Cyclin D1 signaling [75]. The mitigated TROAP effect was demonstrated in mouse xenografts with TROAP-overexpressing HCC cells by blocking Dyrk1B activity using AZ191. Moreover, AZ191 treatment enhanced the cytotoxic effect of the chemotherapy drug cisplatin in vivo. Combined treatment with cisplatin and the Dyrk1B inhibitor AZ191 effectively inhibited tumor growth in xenograft tumor assays in BALB/c nude mice injected with Hep3B or PLC8024 hepatocellular carcinoma cell lines [75]. Therefore, targeting Dyrk1B activity may be a promising therapeutic approach for HCC patients with high expression of TROAP.

Notably, synergistic effects of Dyrk1B inhibition combined with chemotherapy have been reported in tumor cell killing [44,53,63,75,81]. Dyrk1B inhibition combined with radiotherapy remains to be investigated. In a novel approach [77] aimed at overcoming radiotherapy-related treatment resistance by targeting Dyrk1B kinase activity, the specific Dyrk1B inhibitor AZ191 was used with ionizing radiation (IR) to enhance tumor cell killing under stress conditions in SW620 colorectal cancer cells. Under serum starvation and hypoxic conditions, Dyrk1B protein expression is upregulated in tumor cells, thereby mediating cell cycle arrest as a stress-survival mechanism. To investigate the relevance of Dyrk1B as a stress regulator combined with radiotherapy, colorectal cancer cells SW620 were irradiated under normal and serum starvation conditions with and without AZ191. Treatment with AZ191 resulted in reduced proliferative activity and clonogenicity of SW620 cells alone or in combination with IR, which correlated with DNA damage and increased ROS levels. In particular, AZ191 successfully targeted the hypoxic core of tumor spheroids, while IR preferentially targeted cancer cells under normoxia in the edge of the spheroids. A combined treatment effect was also observed in colorectal carcinoma (CRC) organoids but not in healthy tissue-derived organoids [77]. Hence, the combined treatment of Dyrk1B inhibition and radiotherapy is a promising therapeutic strategy against colorectal cancer [77].

### 3.2. EHT5372 Inhibitor

Deregulation of PI3K/PTEN/Akt/mTOR signaling was observed in solid tumors and in pancreatic and ovarian cancer and plays a crucial role in drug resistance [42,44], indicating mTOR as an attractive drug target. mTOR inhibition alone is not sufficient for the treatment of pancreatic cancer. Inhibition of the mTOR pathway by its inhibitors RAD001, WYE354, or rapamycin resulted in increased Dyrk1B protein levels [44]. Similarly, increased Dyrk1B expression was observed by targeting Akt with AZD5363 or MK-2206 inhibitors. Thus, the pharmacological inhibition of Dyrk1B could be beneficial in cancer therapy targeting mTOR signaling. Indeed, the usage of Dyrk1B inhibitor EHT5372 (Figure 3) enhanced the toxicity of mTOR inhibitor RAD001 in pancreatic Panc1 and ovarian TOV21G, SKOV3, and OVCAR3 cancer cells [44]. Also, combined targeting of Dyrk1B and mTORC1/2, by using both AZ191 and KU-0063794 inhibitors, respectively, resulted in pronounced cytotoxicity in Panc1 cells [42].

Furthermore, in vivo studies revealed the beneficial effect of Dyrk1B pharmacological inhibition by the inhibitor EHT5372. In particular, Panc1 cells were injected subcutaneously in J:NU athymic mice, which were then subjected to intraperitoneal injection with the Dyrk1B inhibitor EHT5372. Administration of EHT5372 in the J:NU mice reduced the size of Panc1 pancreatic cancer xenografts in a dose-dependent manner, suggesting that EHT5372-induced apoptosis predominates the re-entry of cancer cells into the cell cycle. In agreement, in vitro treatment of Panc1 spheroid cells with 10 μM EHT5372 induced apoptosis, resulting in a 13-fold decrease in spheroid size and increased the number of single cells. In addition, the intraperitoneal administration of the inhibitor EHT5372 in Pdx-1-cre LSL/KrasG12D/Ink4a/Arf null B6 mice, a mouse model of pancreatic cancer, resulted in maintenance of their viability for up to 8 weeks, whereas the control mice showed only 30% survival by 8 weeks. Moreover, when co-treated with Dyrk1B inhibitor EHT5372 and mTOR inhibitor RAD001, Pdx-1-cre LSL/KrasG12D/Ink4a/Arf null B6 mice remained viable for 8 weeks, as in the case of EHT5372 treatment alone, but the synergistic effect of co-administration of EHT5372 and RAD001 inhibitors resulted in a 30-fold reduction in pancreatic cancer size and in a reduced number of microscopic tumor foci by 2-fold compared to RAD001 alone [60].

### 3.3. RO5454948 Inhibitor

As mentioned above, Dyrk1B is implicated and highly expressed in pancreatic cancer. Previous studies have demonstrated that Dyrk1B protein levels are elevated up to 10-fold in quiescent G0 cancer cells and that Dyrk1B acts by several mechanisms to block cell proliferation and increase the expression of the antioxidant genes *SOD2* and *SOD3* resulting in reduced reactive oxygen species (ROS) levels and increased cell viability [58,71]. Targeting Dyrk1B activity by the compound **A** (hereafter named RO5454948 inhibitor, see Figure 3), identified from a Roche generic kinase inhibitor library, in the pancreatic cancer cell lines Panc1, SU86.86, and AsPc1 and in the colorectal cancer (CRC) cell line SW620 resulted in increased apoptosis, indicated by cleavage of the apoptotic proteins PARP and Caspase-3, and increased ROS levels. Moreover, DNA damage was observed upon Dyrk1B inhibition, as indicated by increased phosphorylation of the histone protein H2AX. In addition, Dyrk1B inhibition by RO5454948 sensitized the pancreatic cancer cell lines Panc1 and SU86.86 to gemcitabine, as well as the colorectal cancer cell line SW620 to cisplatin [59]. To estimate the potential off-target effect of the RO5454948 inhibitor against Dyrk1A, the closest relative member of Dyrk1B, two colon cancer cell lines were used; SW620 cells, which express both Dyrk1B and Dyrk1A, and HCT116 cells, which express only Dyrk1A, as controls for off-target effects of the RO5454948 inhibitor. Notably, no effect was observed upon treatment of HCT116 cells with RO5454948, suggesting that the beneficial effect of RO5454948 in cancer cell lines is due to Dyrk1B inhibition and not to Dyrk1A [59].

Moreover, the *Dyrk1B* gene was found to be amplified or upregulated in approximately 75% of ovarian cancers [36]. Pharmacological inhibition of Dyrk1B with RO5454948 inhibitor in the ovarian cancer cell lines TOV21G, SKOV3, and OVCAR3 similarly resulted in a selective effect on G0-quiescent cancer cells by promoting cell cycle re-entry, increased apoptosis, ROS levels, and DNA damage [36].

### 3.4. KS40008 Inhibitor

Another study [87] demonstrated a novel mechanism connecting DyrK1A/B function with autophagy in cancer. In particular, a novel inhibitor for Dyrk1A/1B, named KS40008 (4-(3-(4-hydroxyphenyl)-1*H*-pyrazolo [3,4-*b*] pyridin-5-yl) benzene-1,2-diol) (Figure 3) showed potent cytotoxicity toward HCT116 and SW480 colorectal cancer cell lines, stimulated the metabolic reprogramming of HCT116 and SW480 cells by reducing mitochondrial respiration, and induced the mitochondrial and ER stress-dependent autophagy. Moreover, KS40008 displayed strong cytotoxicity to CRC organoids compared with the chemotherapeutic drug 5-FU [87].

### 3.5. DYRKi Inhibitor

Aberrant activation of Hh/Gli signaling was observed in several human cancers [88,89]. Targeting the Hh/Gli signaling with Smoothened (SMO) inhibitors showed remarkable therapeutic effects in patients with advanced and metastatic basal cell carcinoma [41]. Having identified Dyrk1B as a novel drug target for the inhibition of oncogenic Hh/Gli signaling, a Dyrk I class inhibitor, referred to as DYRKi (Figure 3), was used as a potent antagonist of Hh/Gli signaling. In particular, the DYRKi inhibitor efficiently repressed SMO-dependent Gli1 and SMO-independent Gli1 expression in human medulloblastoma cells (DAOY) [41]. Moreover, in vivo inhibition of Dyrk1B by DYRKi impaired the oncogenic growth of Panc1 and L3.6pl xenografts in Foxn1^nu^ nude mice [41].

### 3.6. 108600 Inhibitor 

Triple negative breast cancer (TNBC) remains challenging because of heterogeneous responses to chemotherapy [90]. Limited response to chemotherapy is associated with a greater risk of metastatic progression. Breast cancer stem cells (BCSCs) consist of a chemotherapy-resistant subpopulation responsible for tumor initiation, progression, and metastases. Thus, high-risk patients would benefit from treatments that target chemotherapy-resistant TNBC and enhance chemosensitivity [90]. A novel multi-kinase inhibitor, 108600 (Figure 3), targets Dyrk1A/B, Dyrk2, CK2α1/α2, and TNIK kinases in a dose-dependent manner and exhibits beneficial effects on the TNBC BCSC sub-population. In vitro treatment of triple negative MDA-MB-231 cells and CTG1883 TNBC organoids with 108600 led to reduced growth of colony and mammosphere formation of BCSCs by inducing G2/M arrest and apoptosis. In vivo treatment with 108600 of female athymic (NCr-nu/nu) mice injected with MDA-MB-231 xenografts resulted in decreased tumor volumes [90]. In addition, administration of 108600 in tumor-bearing nod/scid/gamma (NSG) mice, injected with ductal carcinoma TNBC PDX (TM00098) cells, resulted in reduced tumors in vivo without adverse effects on the animals’ body weights and exhibited beneficial effects in TNBC PDX tumor growth combined with paclitaxel chemotherapy. Thus, combinatory treatment with 108600 and chemotherapy suppresses the growth of pre-established TNBC metastases, providing supplemental support for the clinical translation of this inhibitor to clinical trials [90].

### 3.7. Pyrrolopyrimidine Inhibitors—VER-239353 Inhibitor (Compound ***34***)

**Figure 4:**
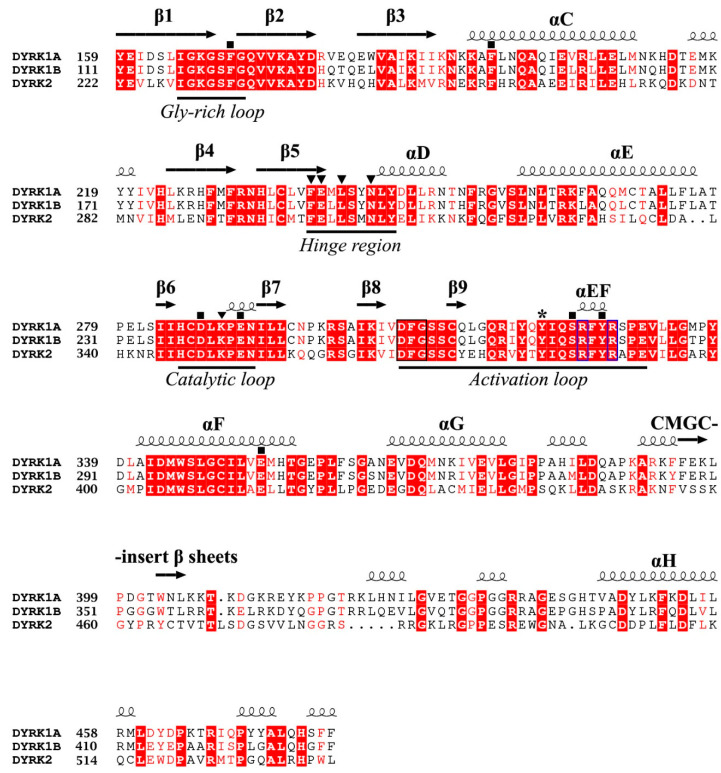
Sequence alignment of the kinase domains of human Dyrk1A (Uniprot: Q13627), Dyrk1B (Uniprot: Q9Y463), and Dyrk2 (Uniprot: Q92630) kinases. The main secondary structure elements and characteristic functional loops/regions are shown. The auto-phosphorylation site on the activation loops of Dyrk1A and Dyrk1Β is shown with an asterisk. Identical amino acid residues are highlighted in red and similar residues are shown in red letters. The Dyrk1A residues making critical (mostly polar) interactions with the bound ATP (see Section 4.2) are indicated with black boxes, whereas the residues interacting with a consensus peptide substrate (see Section 4.3) are indicated with inverted triangles. All of these residues are highly conserved in DYRKs, except Dyrk1A-Asn 244, which is replaced with an aspartic in Dyrk3 (Dyrk 3 and Dyrk 4 are not shown for simplicity). The conserved DFG motif of the activation loop is enclosed in a blue box and the conserved Arg residues interacting with the phosphorylated Tyr (shown with an asterisk) of the activation loop are enclosed in a black box (see Section 4.1). The sequence identity between kinase domains of Dyrk1A and Dyrk1B is 85%, whereas their identity with Dyrk2 is 45%.

By using fragment and structure-based discovery methods, Walmsley and colleagues [79] aimed to identify highly selective adenosine triphosphate (ATP) competitive inhibitors targeting Dyrk1A and Dyrk1B. Fragment-based discovery hits identified multiple series of pyrrolopyrimidine compounds, including the compound **34**, [4-(4-{[(3,5-Difluorophenyl) methyl] amino}-2-methyl-7*H*-pyrrolo-[2,3-*d*] pyrimidin-5-yl) pyridine-2-amine] (Figure 3). The methyl group on the pyrrolopyrimidine of compound **34** was reported to be an important determinant of selectivity for Dyrk1A/Dyrk1B against other kinases. Compound **34**, named thereafter VER-239353 inhibitor, exhibits similar low nM potency against Dyrk1A and Dyrk1B, since Dyrk1A and Dyrk1B vary by only one amino acid in the ATP-binding pocket (Dyrk1B-Leu 192 instead of Dyrk1A-Met 240 at their hinge regions of the ATP-binding pocket; see Figure 4) and exhibits high selectivity versus Dyrk II class despite their similar ATP-binding sites. In particular, VER-239353 exhibits an IC_50_ of 7 nM and 2.4 nM against Dyrk1A and Dyrk1B, respectively, and >30-fold selectivity versus Dyrk2 [79]. Human glioblastoma U87MG cells treated with VER-239353 exhibited increased expression of pRb, cyclin D1, and Dyrk1B, and increased the expression of p21 and p27 cell cycle inhibitors, resulting in G0 to G1 transition. According to that study [91], paradoxically, cell proliferation was inhibited by the induced re-expression of Dyrk1B inhibition, but the cell growth arrest induced in quiescent cells by Dyrk1A/B inhibition was reversible through the addition of growth-promoting factors. Furthermore, Dyrk1A/B inhibition by VER-239353 combined with CHK1 inhibition by VER-157932 induced DNA damage in U87MG multi-cellular spheroids. In vivo, VER-239353 inhibited the growth of U87MG glioblastoma xenografts in Crl:NU(NCr)-Foxn1nu mice [91].

### 3.8. Anilinopyrimidine, Methylated Azaindole, 2-Phenylpyrimidine, and 2-Alkylpyrimidine Inhibitors

In a study [80] aiming to develop potent Dyrk1B inhibitors for combined treatment with MEK inhibitors AZD6244 or AZD8330 in melanoma tumors, a set of potent, selective, and cellular active Dyrk1B inhibitors was developed through a combination of targeted screening and structure-based design, as synthetic derivatives of azaindole (compound **2**). In particular, anilinopyrimidine inhibitors (compounds **12**–**18**), methylated azaindole inhibitors (compounds **19**–**22**), 2-phenylpyrimidine inhibitors (compounds **27**–**30**), and 2-alkylpyrimidine inhibitors (compounds **31**–**34**), were synthesized and subsequently tested for Dyrk1B inhibition, by using in vitro kinase assays, cell assays, kinase selectivity profiles, and in vivo bioavailability studies (Figure 3). Notably, compound **34** described in this study belongs to 2-alkylpyrimidines and is different from VER-239353 (also referred to as compound **34** [79]), as mentioned above (see Figure 3). All described compounds in this study showed increased Dyrk1B selectivity with IC_50_ values ranging from 1 nM (compound **14**) to 98 nM (compound **27**) as determined in cell-free assays. Compounds **19**, **31**, and **33** with Dyrk1B IC_50_ values of 3 nM, 7 nM, and 7 nM, respectively, were furthermore assessed for broader kinase selectivity in a diverse panel of 124 kinases at a concentration of 1 μM, and compound **33** appeared to be the most selective of these, with no other kinases inhibited above the 50% level. Unfortunately, none of these three compounds, when tested in A375 melanoma cells in combination with the MEK inhibitor AZD6244, exhibited a significant increase in cell death compared to AZD6244 alone.

### 3.9. Azaindole-Quinoline-Based Inhibitors 

Given that GSK3β kinase constitutes a natural off-target in the design of selective Dyrk I class inhibitors, the selectivity over GSK3β is one of the major objectives in the development of potent Dyrk1B inhibitors. Regarding this point of view, Szamborska-Gbur and colleagues [92] performed a detailed comparative structural analysis of ATP-binding sites between Dyrk1B and GSK3β, and they identified key regions responsible for selectivity by building and optimizing a homology model taking advantage of comparative modeling and metadynamics simulations in the absence of the Dyrk1B structure at that time. By calculating the interaction energies between docked ligands in the ATP-binding sites of both kinases, they proposed amino acid residues responsible for potency and selectivity. Especially, three amino acid residues located in the ATP pocket of Dyrk1B, such as Phe 190, Val 258, and Glu 243, are crucial for the high affinity and selectivity of both compounds **B** and **C** series belonging to azaindole–quinoline derivatives (Figure 3). These findings support the design of potent and selective Dyrk1B inhibitors based on azaindole–quinoline derivatives. 

### 3.10. Macrocyclic Inhibitors—JH-XVII-10 Inhibitor

Development of the first macrocyclic inhibitors of Dyrk1A/Dyrk1B was recently reported by Powell and colleagues [93]. Based on the JH-XIV-68-3 inhibitor (compound **3**) that displays selectivity for Dyrk1A/Dyrk1B in biochemical and cellular assays, as well as an antitumor efficacy in head and neck squamous cell carcinoma (HNSCC) cell lines, the derivative JH-XVII-10 inhibitor (compound **10**) was generated by the introduction of fluorine to block the 2-position of the azaindole, rendering the molecule resistant to aldehyde oxidase (AO) activity, in which the JH-XIV-68-3 inhibitor (compound **3**) is vulnerable (Figure 3). The JH-XIV-68-3 inhibitor showed an IC_50_ value of 13 nM or 19 nM for Dyrk1A and Dyrk1B, respectively, while the JH-XVII-10 inhibitor has an IC_50_ value of 3 nM or 5 nM for Dyrk1A and Dyrk1B, respectively. The JH-XIV-68-3 and JH-XVII-10 inhibitors were further tested for their selectivity in the HNSCC CAL27 and FaDu cell lines and showed a significant decrease in cell proliferation [93]. Thus, these novel scaffolds present a potential new avenue for therapeutic development targeting Dyrk I class in cancer, but they do not solve the problem of selectivity between Dyrk1A and Dyrk1B kinases. 

### 3.11. 1H-pyrazolo [3,4-b] Pyridine Inhibitors—Compound ***8h***

Park and colleagues [94] reported the synthesis of 1*H*-pyrazolo [3,4-*b*] pyridine derivatives as potent inhibitors of Dyrk1A/Dyrk1B. In particular, a set of diaryl 1*H*-pyrazolo [3,4-*b*] pyridine derivatives (compound series **8** and **9**) (Figure 3) showed excellent Dyrk1B inhibitory enzymatic activities with IC_50_ values ranging between 3 and 287 nM. Among them, the 3-(4-hydroxyphenyl), 5-(3,4-dihydroxyphenyl)-1*H*-pyrazolo [3,4-*b*] pyridine inhibitor (compound **8h**), which exhibits the highest inhibition for Dyrk1B with IC_50_ of 3 nM, showed remarkable cytotoxicity in the colon cancer cell lines RKO, HCT116, DLD-1, SW480, and SW620. In addition, treatment of SW480 and SW620 spheroids with 5 μΜ of compound **8h** for 10 days resulted in cell growth inhibitory effect. Moreover, compound **8h** showed a cytotoxic effect in patient-derived colon cancer organoids after 5 days of treatment, suggesting its potential medicinal value in colon cancer. However, it should be mentioned that all of the produced series of compounds **8** and **9** described in this study (Figure 3) also exhibit specificity for Dyrk1A to some extent.

### 3.12. Lead-Like 2,4-Bisheterocyclic Substituted Thiophenes Inhibitors—Compound ***48***

In a study [95] aiming to discover novel inhibitors for Dyrk I class kinases, a library of lead-like 2,4-bisheterocyclic substituted thiophenes was screened, and the thiophene compound **48** (Figure 3) was identified as a potent and selective Dyrk1B inhibitor [95]. In particular, compound **48** exhibits IC_50_ of 0.1 μM, 0.07 μM, and 0.04 μM for Dyrk1A, Dyrk1B, and Dyrk2 kinases, respectively. In a larger selectivity screen, only Clk1 and Clk4 were identified as additional off-targets of compound **48**. Treatment of the osteosarcoma cell line U2OS with thiophene compound **48** resulted in increased apoptosis and increased ROS levels [95]. 

### 3.13. In Silico Identification of Potent and Selective Dyrk1B Inhibitors

A previous study reported a novel multi-stage compound discovery algorithm, the QSAR algorithm, which aimed at the in silico identification of potent and selective Dyrk1B inhibitors from a large set of initial candidates [96]. The method used structure-based docking and ligand-based quantitative structure-activity relationship modeling based on known crystal structures of Dyrk1A. This approach resulted in the identification of small molecules that target Dyrk1B with high efficiency and specificity. In particular, the QSAR algorithm shortened the optimization cycle to only three iterations on subsets of size <10% each and reduced the required number of compounds to be synthesized by 70%. 

## 4. DYRK Structure, Crystallography Studies, and Inhibitor Selectivity 

To successfully design small compounds with potent inhibitory and highly specific functions, it is necessary to incorporate knowledge accumulated by X-ray crystal structures of DYRKs and homologous proteins. As previously mentioned, DYRKs constitute an evolutionarily conserved family of kinases comprising five members (Dyrk1A, Dyrk1B, Dyrk2, Dyrk3, and Dyrk4). Apart from their kinase domain, they have additional regulatory domains required for their function, localization, and inter-molecular interactions. Their catalytic kinase domain follows the DYRK-characteristic homology (DH) box, comprising an N-terminal lobe (N-lobe) with five antiparallel β-strands and a conserved regulatory αC-helix, as well as a larger C-terminal (C-lobe) consisting of α-helices [97]. Their kinase domain is highly conserved as shown from the sequence alignment of human Dyrk1A and Dyrk1B with Dyrk2, with Dyrk1A sharing 85% identity with Dyrk1B, while both of these kinases present 45% identity with Dyrk2 (Figure 4). To date, many crystal structures of Dyrk1A in its complexes with several natural or synthetic inhibitors have been published (some of which are discussed below), facilitating drug design against Dyrk1A, whereas elucidation of the structure of Dyrk1B has been accomplished only very recently.

### 4.1. Overall Structure of the Kinase Domain of DYRKs and Activation Mechanism

**Figure 5:**
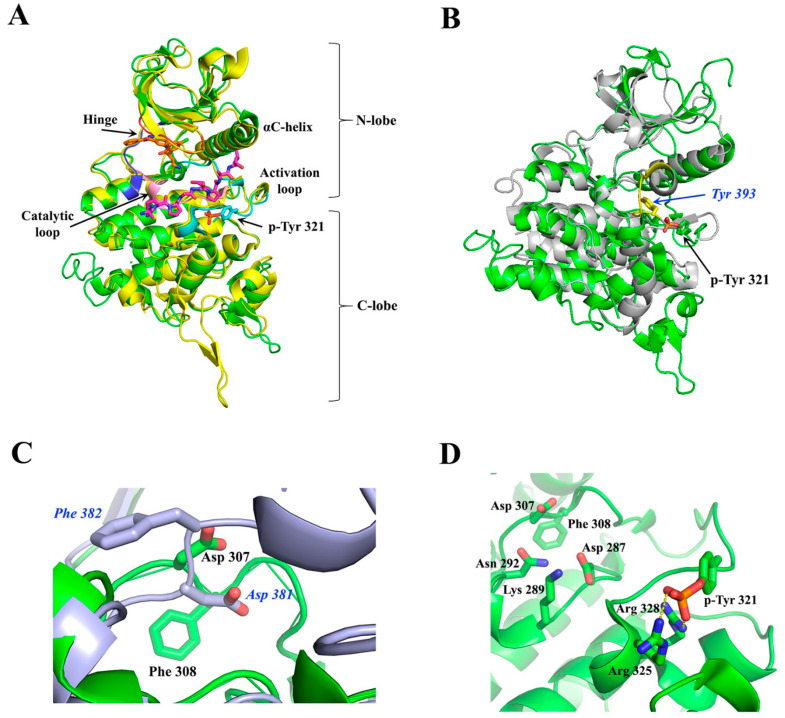
Structural comparisons of activated and non-activated homologous kinases. (**A**) Superposition of Dyrk1A (green) with Dyrk2 (yellow). Dyrk1A (PDB: 2WO6) [98] is in complex with the consensus substrate peptide RARPTPALRE (in magenta) and with the ATP inhibitor DJM2005 (in orange), whereas Dyrk2 is in its Apo-form (PDB: 3K2L) [98]. The hinge region and the activation and catalytic loops of Dyrk1A are colored dark blue, light blue, and pink, respectively. (**B**) Superposition of the activated Dyrk1A (PDB: 2WO6; green) with the corresponding inactivated Abl kinase domain (PDB: 2HYY; light grey) [100]. The autophosphorylated Tyr 321 (p-Tyr 321) of Dyrk1A and the equivalent non-phosphorylated Tyr 393 of Abl (in blue italics) are shown as sticks. The section of the activation loop of Abl, colored in yellow, adopts the “inactive conformation”, occupying the substrate binding cleft. (**C**) Close-up views of the DFG-in and DFG-out motifs in Dyrk1A (PDB: 2WO6; green) and Abl (PDB: 2HYY; light grey), respectively. Dyrk1A residues are shown in black, whereas Abl residues are in blue italics. (**D**) Close-up view of the activation loop and of the substrate binding cleft of Dyrk1A (PDB: 2WO6). The polar interactions between p-Tyr 321 and Arg residues 325 and 328, locking the activation loop in its “active conformation”, are shown. Also shown is the DFG-in conformation of Dyrk1A, where Asp 307 together with Asn 292 can co-ordinate Mg^2+^ for the hydrolysis of the bound ATP molecule, and the orientation of the catalytic Lys 289 which catalyzes the phosphoryl transfer reaction from the ATP to the substrate. Images were created with PyMOL 1.8.0.4 version.

The crystal structure of the ternary complex of Dyrk1A bound with an ATP-competitive inhibitor (DJM2005) and a consensus peptide substrate RARPTPALRE [98] showed that ATP binds at a specific region between the N- and C-lobes of the kinase domain, connected by a hinge region, called the ATP-pocket or cleft, whereas the substrate binding site is in close proximity to the ATP pocket, lying in a region flanked by their catalytic loop, αC helix, and activation loop (Figure 5A). The activation loop is located at the C-lobe (Figure 5A) extending from the conserved DFG motif (Asp 307, Phe 308, and Gly 309, Dyrk1A numbering) to the also conserved RFYRSPE sequence (Figure 4). As discussed previously, DYRKs rapidly auto-activate during their folding process by autophosphorylation on the second tyrosine residue of the conserved activation loop YxY motif (Tyr 321 of Dyrk1A or Tyr 273 of Dyrk1B) (Figure 4 and Figure 5A) and subsequently abolish their tyrosine phosphorylation ability, while retaining only serine/threonine phosphorylation ability [99]. Phosphorylation of this conserved tyrosine leads to a “locked” re-orientation of the otherwise loose and flexible activation loop in such a way that the substrate binding site is formed, as is shown by the superposition of the activated Dyrk1A with the structure of the non-phosphorylated and inactivated human Abl kinase (Figure 5B). 

In the literature [98,100], the active conformation of kinases is often referred to as “DFG-in” conformation, since the conserved Asp 307 in Dyrk1A (Asp 259 in Dyrk1B) is pointing inwards the catalytic cleft (Figure 5C), while “DFG-out” corresponds to their inactive state, where the “unlocked” activation loop occupies the cleft for substrate binding, as for example is shown for the inactive Abl kinase upon its superposition to the constitutively active Dyrk1A (Figure 5C). In the DFG-in motif, the aspartate forms contact with the phosphate groups of the bound ATP either directly, or through magnesium. The phenylalanine in DFG is responsible for the hydrophobic interaction with the αC-helix and the correct orientation of aspartate in the DFG motif. The locked, or otherwise activated, conformation of the activation loop is governed by polar interactions of the phosphorylated tyrosine with two highly conserved arginine residues, Arg 325 and Arg 328 (Dyrk1A numbering), located at the end of the activation loop (Figure 5D). Thus, in contrast to other kinases, mature DYRKs are always activated, and their overall structure is the same, regardless of the binding of ATP or substrate to them, as shown by the superposition of the crystal structure of the Apo-form (free-state) of Dyrk2 with the Dyrk1A with a bound ATP analogue (ADPNP) and a substrate peptide (Figure 5A).

### 4.2. ATP-Binding Site of DYRKs 

**Figure 6:**
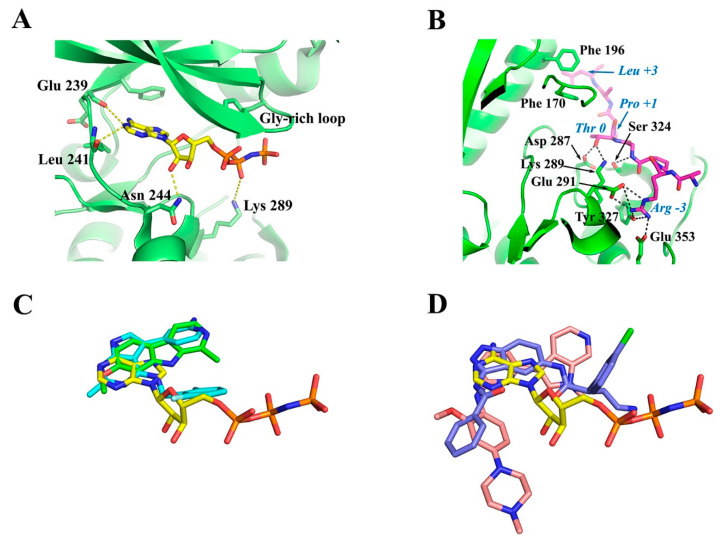
ATP- and substrate-binding sites of Dyrk1A. (**A**) Close-up view of ADPNP (ATP analogue) bound to the ATP-binding site of Dyrk1A (PDB: 7A4O) [79]. The polar interactions between Dyrk1A and ADPNP (stick representation) are only shown as dashed lines. (**B**) Close-up view of the interactions of the consensus substrate peptide RARPTPALRE with Dyrk1A (PDB: 2WO6) [98]. Polar interactions are shown as dashed lines. The Dyrk1A interacting residues are in black, whereas the residues of the peptide are in blue italics. The numbering of the peptide residues starts at 0 for the threonine residue which is due to phosphorylation. (**C**,**D**) Superposition of small (**C**) and large (**D**) ATP-competitive inhibitors with ADPNP as in their crystal structures with Dyrk1A. The adenosine moiety of ADPNP is in yellow and its phosphate groups are in orange. In panel (**C**), the natural inhibitor harmine is shown in green (PDB: 3ANR) [97] and the VER-239353 inhibitor in cyan (PDB: 7A5N) [79]. In panel (**D**), the AZ191 inhibitor is shown in light pink (PDB: 8C3G) [101] and the DJM2005 inhibitor (PDB: 2VX3) [98] in deep blue. Images were created with PyMOL 1.8.0.4 version.

Interestingly, the crystal structure of Dyrk1A with an ATP analogue (ADPNP) has also been solved [79], revealing the interactions of a bound ATP molecule with the ATP cleft. As shown in Figure 6A, the adenosine moiety of ADPNP is buried in a hydrophobic pocket with the phosphate backbone oriented towards the solution packed below the glycine-rich loop. The glycine-rich loop comprises a GxGxxG motif (G_166_KGSFG in Dyrk1A and G_118_KGSFG in Dyrk1B; see Figure 4), constituting a part of ATP pocket from the N-lobe. An important residue, located at the beginning of the hinge region in the ATP-pocket of DYRKS, is the so-called “gatekeeper” phenylalanine residue (Phe 238 in Dyrk1A and Phe 190 in Dyrk1B) which confers selectivity to inhibitors in DYRKs among other kinases (Figure 6A). The most critical residues making polar interactions with the bound ATP molecule (Figure 6A) are highly conserved in DYRKs (Figure 4), which makes it a challenge for the development of selective ATP-competitive inhibitors for a specific kinase. Of course, there are many other hydrophobic interactions with the bound ADPNP molecule (not shown in Figure 6A), which are also conferred by highly conserved hydrophobic residues from parts of the Gly-rich loop, the catalytic loop, and the hinge region that flanks the ATP pocket.

### 4.3. Substrate-Binding Site of DYRKs

The substrate binding site of DYRKs has also been revealed in detail as in the case of the crystal structure of the ternary complex of Dyrk1A with the consensus substrate peptide RARPT*PALRE (asterisk denotes the threonine residue to be phosphorylated) and the ATP-competitive inhibitor DJM2005 (PDB ID: 2WO6) [98]. This study showed that Dyrk1A phosphorylates substrates exclusively on Thr or Ser residues on peptides that have proline at the P+1 position (P denotes the phosphorylation site), explaining why DYRKs have been considered to be proline-directed kinases. However, the study also showed that preference for the P+1 site is not exclusively limited to proline, since some DYRKs can also accommodate small hydrophobic residues (e.g., Gly, Ala, Val) at this site. Another notable preference observed was arginine at position P−3 of the substrate, poised for occupying the C lobe electronegative pocket (interactions with Glu 291, Tyr 327, and Glu 353), as well Leu at P+3 making hydrophobic interactions with two highly conserved phenylalanine residues (Phe 170 and Phe 196) from the Gly-rich loop and the αC-helix (Figure 6B). Also, the hydroxyl group of the Thr residue of the peptide substrate, which is the target of phosphorylation (designated as Thr 0), was shown to form hydrogen bonds with the highly conserved Asp 287 and Lys 289 of the catalytic loop and with the invariant Ser 324 of the activating loop of Dyrk1A (Figure 6B). 

As revealed by crystal structures of other GMGC kinases with bound ATP and Mg^2+^ or Mn^2+^ (e.g., cAMP-dependent protein kinase; PDB: 1ATP) [102], the conserved Asp 307 together with the invariant Asn 292 co-ordinates the divalent ion, while the transfer of the γ-phosphate group to the bound substrate is mediated by the catalytic Lys 289 which is in an optimal conformation by its interaction with Asp 287 (numbering is based on Dyrk1A). The spatial orientation of the critical Dyrk1A residues for catalysis of substrate phosphorylation is shown in the upper left part of Figure 5D. Taken together, the structural information accumulated by the crystal structures of DYRKs and some homologous kinases shows that apart from the ATP-binding site, the residues that govern substrate recognition (Figure 6B) are also highly conserved, as shown in the alignment among Dyrk1A, Dyrk1B, and Dyrk2 (Figure 4). Thus, development of specific inhibitors targeting the substrate binding site is also a very challenging task.

### 4.4. Development of Dyrk1A/B Inhibitors

To our knowledge, all reported active compounds to date with known crystal complexes with DYRKs target the ATP-binding site of DYRKs, despite the high similarity between them, based on limited subtle differences that occur at these sites to achieve selectivity. It should also be noted that when designing selective Dyrk1A/B inhibitors, natural off-targets of the GMGC family are also taken into account to avoid undesired side effects.

In general, kinase inhibitors are mainly classified into three types: Type I refers to ATP-competitive inhibitors that block the kinase through binding at its ATP-binding domain. Types II and III are non-ATP-competitive inhibitors and both bind to allosteric druggable pockets that are present in the DFG-out conformation. The difference between them is that type II inhibitors bind both to a part of the ATP-binding site and to the nearby allosteric pocket available in the inactive kinases, whereas type III inhibitors occupy exclusively the allosteric pocket [100,103,104,105]. Apparently, in the case of DYRKs, which are constitutively active (DFG-in conformation), the development of type I inhibitors has attracted the interest of the most researchers, leading to a plethora of highly potent Dyrk1A inhibitors, and in various cases to their crystal structures with Dyrk1A, as elegantly reviewed recently [106].

However, it has been shown that for constitutively active kinases such as DYRKs, a functional transitional intermediate exists during their translation with a probable different structure to that of the mature kinase, which is capable of autophosphorylation of the activation-loop tyrosine [99]. Moreover, this pioneering study showed that this transitional intermediate can be targeted by small molecules preventing its autophosphorylation. Interestingly, a selective inhibitor of the transitional-intermediate of Dyrk1A was then discovered, named FINDY [107], which inhibited the Dyrk1A folding process by preventing its autophosphorylation on Ser 97 with an IC_50_ value of 110 nM, whereas it did not affect Dyrk1B and Dyrk2. Whether the FINDY-targeted folding intermediate of Dyrk1A resembles an inactive DFG-out conformation of Dyrk1A remains to be answered. 

During the last decade, following the first crystal structure of Dyrk1A with harmine [97], a potent natural Dyrk1A inhibitor with reported IC_50_ values in the range 30–80 nM [108,109], several studies have been published based on X-ray crystallography and sometimes in combination with NMR to identify the binding motifs of fragments; indeed some hits from such fragment screening analyses have been optimized to highly potent compounds for Dyrk1A and in some cases with enhanced selectivity against off-targets [110]. For example, through such structural approaches, novel harmine-derived or synthetic compounds with high selectivity on Dyrk1A/B were discovered, as well as new scaffolds for Dyrk1A/B inhibition, including biaryl compounds and imidazopyridine compounds [79,97,111,112,113,114]. One such promising compound discovered by fragment screening and X-ray crystallography is the VER-239353 inhibitor (Figure 6C), a highly selective, well-tolerated Dyrk1A/B inhibitor which was discussed in more detail above [79]. 

The common feature of these studies is that they all deal with ATP-competitive inhibitors binding to the canonical binding site, but occupying different volumes inside this site and in some cases introducing novel interactions with the cleft apart from those observed in ATP analogues (Figure 6C, D). In the absence, until very recently, of a crystal structure of Dyrk1B, homology models have been used to develop specific inhibitors for this kinase. Following such approaches, the design of some potent and selective azaindole-quinoline-based Dyrk1B inhibitors has been reported, as previously mentioned [92]. 

### 4.5. Dyrk1B Crystal Structure 

**Figure 7:**
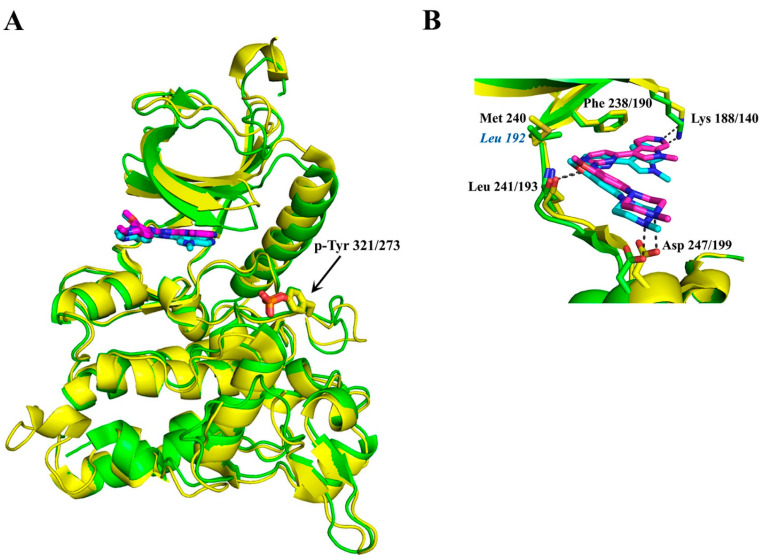
Structural comparison of Dyk1A and Dyrk1B kinase domains. (**A**) Superposition of the AZ191-bound kinase domains of Dyrk1A (yellow; 8C3G) and Dyrk1B (green; 8C2Z) [101]. The bound AZ191 is shown in magenta or cyan for Dyrk1A or Dyrk1B, respectively. The phosphorylated tyrosine residues 321 or 273 of Dyrk1A or Dyrk1B, respectively, are shown in sticks on their activation loops. (**B**) Close-up view of important and identical polar interactions between AZ191 and Dyrk1A or Dyrk1B. The only difference in the ATP-binding site of these kinase domains is one residue in the hinge region (Dyrk1B-Leu 192 instead of Dyrk1A-Met 240). Images were created with PyMOL 1.8.0.4 version.

Interestingly, while this review was under preparation, the first crystal structure of human Dyrk1B in its complex with the small ATP-competitive inhibitor AZ191 appeared (PDB ID: 8C2Z), together with the structure of a similar complex with the highly homologous Dyrk1A (PDB ID: 8C3G) (Figure 7A) [101]. As expected, due to the highly conserved ATP-binding pocket and the similar affinities of these kinases to AZ191 (discussed in Section 3.1), the binding motif of AZ191 to Dyrk1A and Dyrk1B is almost identical (Figure 7B), justifying the quite similar potencies for Dyrk1A and Dyrk1B as mentioned previously. However, these two binding sites differ only in one non-interacting residue at the hinge; instead of a Leu residue at position 192 in Dyrk1B, a larger Met residue exists in Dyrk1A at position 240, whose longer side-chain could come into closer proximity with the bound molecules. Therefore, appropriate modifications at the phenyl ring of AZ191 could lead to a modified molecule with enhanced selectivity between these highly similar kinases.

### 4.6. Current Limitations and Future Perspectives in the Development of Dyrk1B Inhibitors 

The ATP-binding site is highly conserved in DYRKs, making it a challenge for the development of selective ATP-competitive inhibitors for Dyrk1B. Moreover, amino acids that govern substrate recognition in Dyrk1A, Dyrk1B, and Dyrk2 kinases are also highly conserved, rendering the design of selective non-ATP-competitive inhibitors for Dyrk1B difficult as well. Another main limitation in the design of selective Dyrk1B inhibitors has been its unknown crystal structure. Remarkably, a crystallizable construct of Dyrk1B was reported recently, together with its crystal structure in its complex with AZ191 inhibitor [101]. Thus, fragment screening of libraries by using this Dyrk1B construct is now feasible to identify novel hits that could serve as a scaffold for the design of highly selective Dyrk1B inhibitors. Also, a good strategy would be the use of a crystallizable complex of Dyrk1B with an ATP analogue for performing in crystallo fragment screening to identify druggable pockets which could accommodate highly selective allosteric non-ATP-competitive inhibitors. In parallel, a series of modifications in already reported ATP-competitive inhibitors targeting Dyrk1B could lead to novel molecules with enhanced selectivity. 

## 5. Conclusions

Mirk/Dyrk1B kinase is a negative regulator of the cell cycle maintaining the survival of quiescent cancer cells and conferring resistance to chemo- and radio-therapy approaches. Pharmacological inhibition of Dyrk1B leads quiescent cancer cells to re-enter the cell cycle, rendering them vulnerable to conventional cancer treatments, induces apoptosis in cancer cells, and reduces tumor size in vitro and in vivo. As Dyrk1B has been established as a pharmacological target in cancer therapy, the discovery of Dyrk1B-specific and potent inhibitors has become even more desired and highly needed. Most of the Dyrk1B inhibitors reported to date are proven effective in cancer treatment, but still exhibit off-target effects on other GMGC or DYRK kinases, and mainly on Dyrk1A, the closest member of Dyrk1B. Interestingly, AZ191, EHT5372, DYRKi, 108600, and VER-239353 inhibitors exhibit clinical relevance, providing a perspective for entering clinical trials. Accumulating X-ray crystal structures of complexes of Dyrk1A with various inhibitors together with the recently accomplished elucidation of the crystal structure of Dyrk1B open the way for designing highly selective Dyrk1B inhibitors.
